# Medical Opinion and Movement

**Published:** 1909-07-24

**Authors:** 


### Medical Opinion and Movement.

THE Treatment of Pneumonia by Iron is discussed in the *Indian Medical Gazette* by Capt. Thompson, I.M.S., who has treated a series of cases in the Midnapore Central Gaol with a modification of Basham's prescription. Every four hours he gave a mixture containing liq. ferri perchlor., ♏xv; liq. ammon. acetat., ʒj; aq. chloroformi, ad ℥j. No other drug or stimulant, except a brisk purge at the commencement, was given in ordinary cases; but any signs of cardiac failure or embarrassment were treated by the early exhibition of strychnine and digitalis. Alcohol was given to tide over any period of collapse at the crisis, if it seemed necessary. Until the adoption of this iron treatment the mortality in the gaol had always been very high—even up to 77 per cent.—owing to the poor physique of the patients; but since trying this routine the mortality from pneumonia has been but 9 per cent. The author recognises that many cases of pneumonia will do well on any treatment, but he believes also that iron exerts an especially favourable influence on the disease, and is preferable to the excessive use of stimulants often adopted. It has not, he admits, any specific action against the pneumococcus; but by increasing hæmoglobin it aids oxygenation, and thus helps the heart, so carrying out one of the main indications in the treatment of pneumonia.

---

THE Endemic Fevers of the Mediterranean Littoral are no longer, since the brilliant successes of Bruce and subsequent investigators into the once mysterious Malta fever, lumped together under the terms "simple continued" fever, "Mediterranean" fever, and so on; but are being gradually disentangled, and will probably some day be recognisable clinically as several distinct and separate diseases. In the *Journal of the Royal Army Medical Corps* Lieut.-Col. Gerrard attempts to differentiate three kinds of fever prevalent among our garrison in Malta, and usually returned on the sickness sheets as simple continued fever. These three varieties he names provisionally three-day, seven-day, and ten-day fever respectively, and he is inclined to identify the second of these with the disease described under that name by Major Rogers in Calcutta. This seven-day fever begins somewhat gradually, and patients admitted to hospital generally give a history of increasing malaise for two or three days previously: they complain usually of headache and backache. Constipation is the rule, abdominal distension is uncommon, the pulse is slow. Rose spots are often found, but appear as a rule only one at a time: the patient usually gets rid of his aches in a day or two, and his temperature reaches normal by lysis in about seven days. Malaria parasites are not found in the blood, and Widal reactions to typhoid and paratyphoid cultures are negative. The author confirms the observation of Major Rogers as to the saddle-back appearance of the temperature-chart in the early stages of the illness.

THE three-day fever is that which is commonest, and it presents many features in common with the disease described by Doerr among the Austrian troops on the Adriatic coast. The author believes that he has seen the same fever also in the Punjab, the Transvaal, and Natal. The symptoms come on rather suddenly, and consist of malaise and backache, which subside quickly under the influence of calomel and diaphoretics. A single rose spot is said to occur occasionally in the region of the umbilicus, which becomes a tiny pustule and then dries up. Ten-day fever, on the other hand, is more insidious in onset than either of the others. Headache is often severe, backache in not complained of. The flushed cheek, glistening eye, and dry, quivering lip all make one think of enteric fever, says Lieut.-Col. Gerrard. Distension of the abdomen is common, rose spots are seen much as in seven-day fever. The bowels are constipated, the pulse slow, soft, and often dicrotic. Some of these cases agglutinate the bacillus paratyphosus, and the author is inclined to regard all three fevers as clinical entities caused by organisms of the same type as the bacillus of Eberth; the arithmetical progression observed in their duration is, in his opinion, suggestive of relation between the organisms. As for the paths of infection, a mosquito, sand-fly, or house-fly is thought to be the probable channel for the three- and seven-day fevers. The recent work of Doerr and Taussig has, as a matter of fact, established that the Dalmatian three-day fever is carried by a biting sand-fly, which becomes infective eight days after acquiring the blood of a patient; and on this ground and because it can pass through a fine-grained filter, the Austrian observers have suggested that the organism is an ultra-microscopic protozoon.

---

ON occasions which, though very rare, do happen now and then in practically every hospital, the very grave disaster of Post-Operative Tetanus draws attention to the fallibility of the most carefully devised systems of asceptic technique. In the *Dublin Journal of Medical Science* Mr. L. G. Gunn gives a most interesting account of an outbreak in a private hospital, consisting of three cases within nine weeks, preceded nine months before by a suspicious case the exact nature of which is doubtful. The most elaborate and painstaking research was undertaken to trace out the origin of the infection. First and foremost the catgut was suspected, but, though tested thoroughly after each case, was always reported sterile. Moreover, the gut used was from four different consignments, each of which was distributed also to other hospitals without any ill-effects. Incidentally it appears that when the popular Fowler method of sterilising catgut by boiling in alcohol in a Jellett's steriliser (after washing in ether and soaking for some days in alcohol) is adopted, the boiling must be for at least thirty minutes. Dry heat and kumol is said to be even more effective. The theatre had been twice repainted during the year: scrapings from all parts of it were examined, with negative results. The air, sponges, and dressings were each considered in turn, and excluded after careful cultural experiments. Last of all Mr. Gunn investigated the water supply. In a cistern under the roof, installed in connection with the hot-water supply two months before the first (doubtful) case, he found growing several masses of red fungoid material under a crack in the boards which covered the cistern. The conclusion arrived at is that the presence of these fungi and the darkness formed a suitable nidus for some stray tetanus bacillus, and that the occasional running dry of the cistern led to spore formation. The water used in the theatre was always boiled for two hours, all except some which was made up into saline solution, which was boiled for twenty minutes only. It is this saline which is believed to have contained tetanus spores undestroyed. The paper is most instructive throughout, and will repay the attention of all who are interested in the preparation of ligatures and other operating-room requisites.

At a recent meeting of the Société de Biologie, Madame Girard Mangin made an interesting communication on the Toxicity of Extracts of Cancerous Growths. She finds that extracts from cancerous tumours, which have not undergone any suppuration and have been treated aseptically, show the presence of toxic substances when injected into animals. The tumours experimented with were obtained from animals and also from the human subject. The effects of these toxic substances are to reduce the blood-pressure and body temperature. Sometimes paralysis ensues, and, administered in fatal doses, they give rise to convulsions. Death ensues by cessation of respiration before the heart stops. In some cases the animals died in a state of profound anæmia and cachexia without any definite lesion. The extracts were made by pounding the tumour in normal saline solution. The author suggests that the toxicity of a tumour determined in this way may be of greater clinical value than histological examination, and gives examples of cases in which a favourable prognosis, founded upon the non-toxic effect of the tumour, has been confirmed by the subsequent history of the case. Thus in two cases of vegetating and rapidly growing cancers of the breast, extracts failed to show any toxic effect, and there has been no recurrence for three years. Similarly, in a case of renal epithelioma of a child a favourable prognosis has been confirmed.

An interesting discussion took place at a recent meeting of the Society of Internal Medicine of Berlin on the Solubility of the Urates in the System. Dr. Gudzent claims to have demonstrated experimentally that uric acid may form with sodium two different combinations, one, which he calls *lactame*, is relatively soluble in blood serum, while the other, *lactime*, is much less so. He points out that the solubility of urate of soda is diminished by the presence of the sodium ion, and thinks that the fact that cartilage contains more sodium salts than other tissues explains the predilection of this tissue for local deposits of urates. A further observation of the author may prove of some therapeutic importance. He finds that the emanations of radium retard the transformation of *lactame* into *lactime*, and, if sufficiently active, even increase the solubility of *lactame*. In the discussion which followed, Dr. Hans Kohn expressed the view that a more plausible explanation for the predilection of cartilage for urate deposits is to be found in the deficient blood supply, and consequent slow metabolism, which more readily allows the transformation of soluble urates into the less soluble form. This explanation is also applicable to the tophi which are found in extravasations, in the ear and other parts.

Professor Greeff, of Berlin, believes he has discovered the long sought for causal microbe of Trachoma, and gives an account of his research in the *Deutsche Medizinische Wochenschrift*. It is a round or oval-shaped coccus, occurring in clumps, and is smaller than any coccus previously known. It is coloured violet or reddish with Giemsa stains, weakly with the aniline dyes, and not at all by the Gram method. The cocci are present within the epithelial cells, and in the stringy mucus. The clumps are frequently surrounded with a halo. He has not found these bacteria in any other pathological condition but trachoma. In order to find the cocci, a little of the superficial conjunctival epithelium is scraped off and spread upon a cover glass. It is necessary to obtain a fresh, untreated case of trachoma, for as soon as treatment has commenced the germs tend to disappear. After fixing the preparation in absolute alcohol for twenty to thirty minutes a stain of the following preparation is applied for nine hours: Twelve parts of Giemsa-eosin solution, three parts of Azure I., and three parts of Azure II. So far the author has not carried out any cultural or experimental observations, but he is convinced that the cocci he describes are the causal germs of the disease. Further confirmation of these observations will be awaited with interest.

One of the recognised difficulties in Skin Grafting is to retain the skin grafts in position by a suitable dressing, so that the wound can be observed from time to time without risk of interfering with the grafts. For this purpose, Dr. J. S. Davis, of Baltimore, uses coarse netting impregnated with gutta-percha. Dr. Ralph St. J. Perry, of Farmington, recommends silk veiling, sterilised and saturated with iodized paraffin. He gives an account of his method in the *American Journal of Surgery*. The silk netting should have a mesh of $\frac{1}{8}$ to $\frac{1}{4}$ inch, and is spread upon and fastened to wire frames about 6 inches square. In this condition it is boiled for half an hour in water to get rid of any stiffening substance, and then for another half-hour in 1 in 5,000 cyanide of mercury solution. After this it is dried in an oven for five minutes, then saturated with the paraffin solution and dried in the open air. The paraffin solution is made by dissolving boiled paraffin in redistilled gasoline, to which is added some resublimed iodine or iodoform. The paraffin solution rapidly permeates the fibres of the netting, and on evaporation of the gasoline leaves a soft, flexible, non-absorbent, non-adhering antiseptic dressing, through which the secretion of the wound readily passes. Over this gauze or other absorbent dressings can be applied without fear of their pulling off the partially adherent grafts, and the open-mesh allows of easy inspection and cleansing of the wound surface. The author recommends keeping such prepared netting for future use by laying it between sheets of sterile paper kept moist with a solution of cyanide of mercury.

---

The Chalk-like Deposits that appear upon the ears, about the knuckles, and in the olecranon bursa in gouty subjects are usually regarded as consisting almost entirely of sodium urate. That they are composed chiefly of the latter substance is true enough, but it is a new point of considerable interest that they contain not a little cholesterin as well. What the significance of this may be is not yet known, but the discovery may serve to throw new light upon the pathology of this form of gout. In the *Pharmakologische Centralblatt*, Matthes and Ackermann record the following analyses of a large whitish gouty deposit: Moisture 46.7, inorganic matter 11.6, organic matter free from ash 41.7 parts per cent. The ash gave 92.3 per cent. of anhydrous sodium carbonate, 4.8 per cent. of sodium chloride, the remainder being made up chiefly of iron potassium and phosphates. The dry material, organic and inorganic together, contained 76.7 per cent. of sodium urate and 6.8 per cent. of cholesterin.

---

The Bruit de Moulin, or Mill-Murmur, first described by Bricheteau and popularised in France by Morel Lavallée, is considered a characteristic sign of pericardial effusion. It is, however, admitted by those who follow the classical description that the co-existence of such a murmur with tympany of the precordial area justifies a diagnoses of pneumo-pericarditis. At the same time Reynier has pointed out that such a bruit may be entirely extra-pericardial, *e.g.*, when it follows a penetrating wound of the chest-wall with formation of an extra-cardial hydro-pneumothorax, and the prognosis is then much less grave than when its origin is intra-pericardial. If the murmur be due to such a wound, it is audible when the patient is in a horizontal position, but is modified or even disappears when he sits up. These changes do not take place when the murmur is due to pericarditis with effusion. Leriche has just published a case in the *Lyon Chirurgical* which confirms the researches of Reynier, the bruit being audible when the patient lay on his back, but disappearing when he sat up. This point in differential diagnosis would seem important both from the point of view of the surgeon and the patient, since it may prevent a useless operation being performed.

---

A new clinical sign of importance in cases of Injury to the Knee is described by Thooris in a recent number of *la Caducée*. The author calls it the "Sign of Patellar Escape." The patient to be examined is made to rest his leg, with the muscles in a flaccid condition, on a couch, and the patella is then seized between the thumb and middle finger in such a way that the former lies above the base and the latter below the apex of the bone. The patient is then told to make a movement as though to extend the limb. If the limb is healthy the patella is found to slip immediately from the grasp, however powerful this may be, the ligament and tendon below and above the patella taking on the consistency of bone to the touch. If, however, the limb be injured by a blow or strain the patella escapes slowly from the grasp or may even remain held by it. A large series of conditions exists between absolute impotence of the quadriceps femoris as evidenced by the patella remaining held in the grasp, and muscular integrity which is shown by the escape of the bone. The sign, therefore, may be considered an adequate expression of the functional condition of the quadriceps femoris, on which depend the prognosis and treatment of traumatic synovitis. For the past six years, the author, who is medical officer to a cavalry regiment, has treated this condition by mobilisation of the muscles, the movements used, however, being those of extension not flexion. The movements are carried out at first lying down, but at a later period standing up, when the muscle has regained its functional activity. A complete cure requires rather over a fortnight of treatment. The author believes that if his method were generally adopted the number of days of sick leave necessitated by hydrarthroses and hæmarthroses in the French Army could be reduced by about two-thirds, for he has found that in the last six years it has never been necessary to send a case of synovitis of the knee to hospital. A cure always resulting from treatment by this method in the regimental infirmary.

---

In The Hospital of May 29 last we gave an account of Surgeon Wright's Treatment of Tuberculosis by intra-muscular injection of succinimide of mercury. A recent number of the *Lancet* contains the report of a trial of this method on thirteen consumptives, male and female, made by Dr. Squire and Mr. Kilpatrick, at the Mount Vernon Hospital, Hampstead. They conclude that on the whole the patients so treated made quicker progress than the others. No bad results were noticed, or at least only temporary and slight inconvenience, although the experience was naturally not altogether grateful to the subjects of it. The full course of treatment, it will be recalled, is two series of thirty injections each (given at the rate of one every other day), separated by a fortnight's rest. The period of time required is therefore more than six months for a proper trial, and it lessens the value of Dr. Squire's and Mr. Kilpatrick's paper that the longest duration of treatment of any of their cases was eight weeks. The dosage of the mercury salt (gr. $\frac{1}{5}$) is rather less, too, than is laid down in the latest advices from Surgeon Wright, while that author also now dispenses, we believe, with the use of potassium iodide. Most will concur with the opinion expressed in the article, that the treatment deserves extended trial.

